# Effects of Surface Preparation Methods on the Color Stability of 3D-Printed Dental Restorations

**DOI:** 10.3390/jfb14050257

**Published:** 2023-05-05

**Authors:** Zbigniew Raszewski, Katarzyna Chojnacka, Marcin Mikulewicz

**Affiliations:** 1 SpofaDental, Markova 238, 506-01 Jicin, Czech Republic; zbigniew.raszewski@envistaco.com; 2 Department of Advanced Material Technologies, Faculty of Chemistry, Wroclaw University of Science and Technology, Smoluchowskiego 25, 50-372 Wroclaw, Poland; katarzyna.chojnacka@pwr.edu.pl; 3 Department of Dentofacial Orthopaedics and Orthodontics, Division of Facial Abnormalities, Wroclaw Medical University, Krakowska 26, 50-425 Wroclaw, Poland

**Keywords:** 3D printing, dental restorations, color stability, surface preparation, polishing, varnishing, red wine, coffee

## Abstract

Background: Color stability is a crucial performance parameter for dental restorations, and limited research exists on how surface preparation methods affect it. The purpose of this study was to test the color stability of three resins intended for 3D printing, which can be used to make dentures or crowns in A2 and A3 colors. Materials and Methods: Samples were prepared in the form of incisors; the first group was not subjected to any treatment after curing and washing with alcohol, the second was covered with light-curing varnish, and the third was polished in a standard way. Then, the samples were placed in solutions of coffee, red wine, and distilled water and stored in the laboratory. After 14, 30, and 60 days, color changes were measured (presented as Delta E) compared to material stored in the dark. Results: The greatest changes were observed for samples that were not polished, then were placed in red wine dilutions (ΔE = 18.19 ± 0.16). Regarding the samples covered with varnish, during storage, some parts detached, and the dyes penetrated inside. Conclusions: 3D-printed material should be polished as thoroughly as possible to limit the adhesion of dyes from food to their surface. Applying varnish may be a temporary solution.

## 1. Introduction

The field of dentistry has seen rapid advancements in the development of dental materials over the past few decades. One of the most important aspects of dental materials is their ability to mimic the natural appearance of teeth. The esthetic expectations of patients have been continuously increasing, leading to a greater demand for dental restorations that can achieve a high level of color stability, biocompatibility, and mechanical properties.

The color stability of dental materials is crucial because it ensures that the restoration maintains its original color and appearance over time. When dental materials are exposed to staining agents, such as coffee or red wine, they can discolor and become unsightly. Patients expect their dental restorations to match the natural appearance of their teeth, and the development of dental materials with enhanced color stability can help achieve this goal.

Three-dimensional printing is increasingly utilized in healthcare due to its ability to create patient-specific restorations with the necessary accuracy and precision. Examples of 3D printing technology applications include implanted heart valves, elements of rib cages, bones, dentures, orthodontic appliances, and the first maxillofacial implants [1].

Three-dimensional printing technology has revolutionized various industries, including dentistry, by offering more efficient and precise methods for fabricating dental restorations [2]. Additive manufacturing techniques, such as stereolithography (SLA), digital light processing (DLP), and selective laser sintering (SLS), enable the production of dental prostheses with complex geometries and intricate structures that would be difficult to achieve through traditional manufacturing methods [3,4]. The use of resin materials in 3D printing has gained popularity in recent years due to their versatility, biocompatibility, and favorable mechanical properties [5,6]. Additionally, 3D printing has proven to be advantageous in terms of reduced chair time, customization, and waste compared to CAD CAM technology [7].

The decreasing cost of professional printers has led to a surge of interest in this technology among dental technicians, particularly in the use of SLA technology with light-curing resins. Three-dimensional printing technology can also be used in the dental office, especially when it comes to making temporary crowns and bridges. This has allowed for a reduction in the number of visits; during one chair time, the patient can have their dental arches scanned. Using databases from the computer, the design of the future temporary restoration is adjusted. While the dentist is grinding the teeth, the dental assistant can 3D-print a temporary crown or bridge. [8].

Loges and Tiberius address the implementation challenges of 3D printing in prosthodontics, highlighting the need for further research and development. Among the key advantages of this technique are the capacity to print multiple prosthetic elements simultaneously, such as crowns, bridges, and removable dentures, and the elimination of intermediate steps like wax modeling and plaster use [9].

Color stability is a critical aspect of dental restorations, as it directly impacts the esthetic outcome and patient satisfaction [10]. Factors such as staining solutions, surface treatments, and resin composition can affect the color stability of dental materials [11,12]. Dental restorations are frequently exposed to staining agents like coffee, tea, and red wine, which may cause discoloration over time [13]. To ensure the longevity and esthetic appearance of dental restorations, it is crucial to investigate the color stability of 3D-printed dental materials and evaluate the effects of different surface treatments.

Color stability is a crucial performance parameter for patients, and as such, new materials entering the market should be tested for this attribute. Studies have identified various beverages, including coffee, red wine, orange juice, and burqa juice, as agents that can alter the color of materials, including 3D-printed elements [8,14,15,16].

However, limited research exists on how surface preparation methods affect color stability and the geometry of printed objects [17,18]. There are various guidelines in the literature on how to cure samples after polymerization in the printer. For this purpose, you can cover the samples with glycerin, use resin, or raise the temperature during polymerization in the light oven.

A change in the color of printed restorations may also occur during their use in the mouth, as a result of changes in the temperature of the food eaten and tooth brushing with toothpastes and brushes [19].

The speed of printing and the thickness of individual layers affects the mechanical properties of the obtained product, which is very important from the point of view of materials used in medicine. Finite Element (FE) simulations are very useful for analyzing this phenomenon [20].

As each material can be cured in various ways, the obtained results may be significantly influenced by factors such as the thickness and shape of the element itself.

Dental technicians and dentists often question the proper method for finishing a prosthetic restoration. Should self-polishing suffice, or should surfaces be protected with varnishes? [21]. The aim of this study is to address this question by determining the color change of three types of resins designed for 3D printing when placed in different media: coffee, red wine, and distilled water, for two months. Based on previous in vitro tests described in the literature, it is assumed that 24 h in the staining solution simulates food intake for 30 days [22]. The 60-day period can successfully indicate how the material will behave throughout its entire service life, assuming that the use of one prosthesis lasts 2–3 years maximum.

The purpose of this study was to test the color stability of three resins intended for 3D printing, which can be used to make removable dentures or crowns and bridges in A2 and A3 colors. The hypothesis put forward at the beginning of this study is that there will be no separation between surfaces prepared in different ways or test solutions.

## 2. Materials and Methods

### 2.1. 3D Printing and Sample Preparation

**Figure 1:**
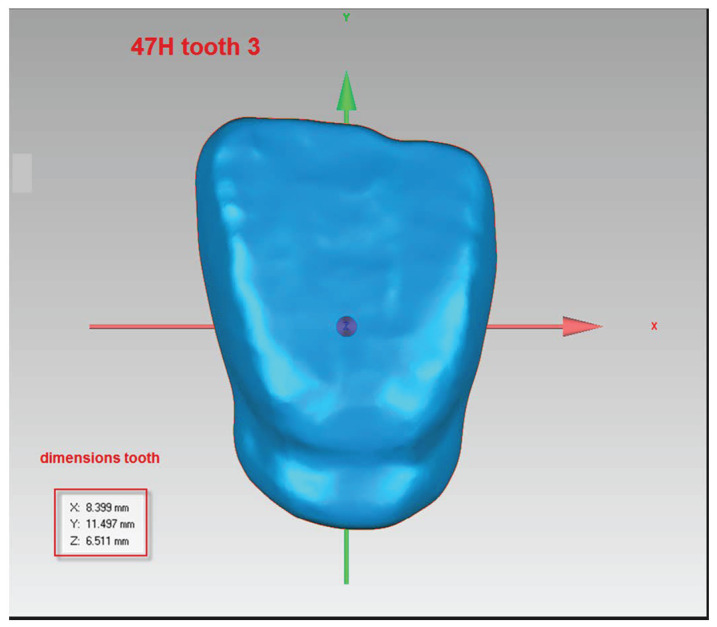
Tooth design before printing in a 3D printer.

Three commercial resins from NextDent were utilized for the tests: Denture 3D+ (dark pink color), Crowntec (color A3), and A2 (NextDent, Soesterberg, The Netherlands). The framework composition of the tested materials based on the available SDS is presented in Table 1. The materials were mixed prior to use with moving rollers for 1 h to ensure proper mixing of the dyes within the resin (according to the manufacturer’s instructions). In total, 141 incisor tooth models were printed, with 47 models for each resin type (Figure 1). The specimens were designed using 3D Builder (10.1.9.0, Microsoft Corporation, Redmont, WA, USA). An STL file of the digital model was uploaded to the software and printed using Liquid Crystal Precision (Photocentric Ltd., Peterborough, UK) with Daylight Polymer Printing technology. The upper central incisors were manufactured layer by layer, with irradiation time for the printing of a single layer of 25 µm thickness being 2 s, and the wavelength being 372 nm. The printing orientation (in a horizontal position) was chosen to maximize accuracy and speed. The position of the samples during printing is very important when it comes to their precision.

When the 3D printer finished its program, objects were removed from the printer building platform with the help of a spatula, and excess resin was cleaned off with a paper towel. After this stage, the samples were coated with glycerin (Sigma Aldrich, Prague, Czech Republic) to protect from the unpolymerized layer, and irradiated in an Evicrobox (SpofaDental, Jicin, Czech Republic) light oven for 10 min, with a power of 1000 watts, and a wavelength of 370 nm. They were then then subjected to an isopropanol bath to remove non-polymerized layers, for 10 min in 98% isopropanol (Sigma Aldrich, Prague, Czech Republic) using a 50 Watt GUC 06A 6L lab ultrasonic washer (Geti, Birmingham, UK). Isopropyl alcohol is effective in cleaning the build plate of the 3D printer and does not leave marks or deposits on the printed elements. The details were discussed in our previous work, Raszewski et al. [23].

### 2.2. Sample Groups

The materials were then divided into four groups:

1. The first group (45 samples) was polished using a 100-micron pumice (EcoPolish Pumice, Goslar, Sliadent, Germany) mixed with water (2:1 ratio) on a WP-EX 2000 II (Wassermann Polishing Units, Ontario Canada) with a felt polishing wheel for 5 min at 800 rpm.
2. The second group (45 incisors) was coated twice with Optiglaze (GC, Tokyo, Japan) varnish and polymerized in the Evicorbox light chamber for 20 min.
3. The third group (45 samples) served as a control and was not processed in any way.
4. The fourth group (6 teeth) was stored in darkness at 23 °C as a reference. Only the 6th sample was selected as a reference sample, because the material is not exposed to factors that can change the color when stored in the dark and at room temperature.

### 2.3. Color Stability Testing

An X Rite spectrophotometer (X Rite, Grand Rapids, MI, USA) measured the initial color of each sample, providing *L*, *a*, *b* values. The color differences were recalculated as Delta *E* using the device’s built-in software (according to Formula (1)). Materials stored under laboratory conditions (group 4) were measured as a reference at the initial step of each measurement in the middle part of the tooth. The resin stored in the dye solutions was measured after the reference tooth from group 4. Each measurement was made 3 times in the middle part of the tooth. Then, the device automatically calculated the average value of the 3 measurements and total color change as Delta *E*:

(1) $$\Delta E=\sqrt{{\left(L1-L0\right)}^{2}+{\left(a1-a0\right)}^{2}+{\left(b1-b0\right)}^{2}}$$

where *L*1 − *L*0—the difference between the brightness of the samples before and after placement in the staining solutions.

*a*1 − *a*0—color change on the red-green axis
*b*1 − *b*0—color change on the yellow-blue axis

Red wine and coffee solutions are standard testing solutions for color changes in prosthetic restorations [11]. A Tschibo Family (Tschibo, Hamburg, Germany) soluble coffee solution and Aguia Moura red wine (Portugal, Douro were prepared. Soluble coffee was prepared by dissolving 2 teaspoons in 200 mL of hot water. For one experiment, 2 L of coffee was prepared. After reaching the temperature of 40 °C, the coffee was divided into 3 PE pots—20 mL. In each solution, 1 sample was placed.

The second test solution was Aguia Moura red wine (Portugal, Douro). After opening the bottle, the contents were poured into PE containers, 20 mL each, in which one sample from the 3 tested resins was placed.

Distilled water was used as the reference solution, into which the rest of the samples were placed.

All the samples were stored at 37 °C in a laboratory drier. Once a week, the solutions were exchanged for new ones. The samples were examined after 14, 30, and 60 days of storage in staining solutions. Before testing, the samples were washed with running water and wiped with a paper towel.

### 2.4. Surface Analysis

During the testing period, material surfaces were analyzed under an optical microscope at 5× magnification (Karl Zeiss, Jena, Germany).

### 2.5. Statistical Analysis

The data were presented as mean and standard deviation (SD). One-way ANOVA was used for statistical analysis. Samples stored in staining solutions were compared with reference samples at 23 °C under laboratory conditions. Statistical analysis was performed with IBM SPSS Statistics for Windows, Version 26.0. IBM Corp. (Armonk, NY, USA). A significance level of *p* < 0.05 was assumed, with time as a variable.

## 3. Results

Table 2, Table 3 and Table 4 present the results of color changes in individual solutions over time for the three tested resins: Crowntec A2, Crowntec A3, and Denture 3D+.

Table 2 shows the color changes of Crowntec A2 resin over time in different media. It is evident that the color change was more significant in red wine and coffee compared to distilled water. Samples without polishing and with lacquer coating showed the most color change in these staining solutions, with *p*-values less than 0.01, indicating a statistically significant difference.

Table 3 displays the color changes of Crowntec A3 resin in various media. Similar to Crowntec A2, the color change was more pronounced in red wine and coffee compared to distilled water. Samples without polishing and with lacquer coating exhibited the most significant color change in these staining solutions, with *p*-values less than 0.01, indicating a statistically significant difference.

Table 4 presents the color changes of Denture 3D+ resin over time in different media. As observed in the previous tables, the color change was greater in red wine and coffee compared to distilled water. Samples without polishing and with lacquer coating demonstrated the most substantial color change in these staining solutions, with *p*-values less than 0.01, indicating a statistically significant difference.

The results indicate that the color changes in the tested resins are more significant when exposed to red wine and coffee than when exposed to distilled water. The samples without polishing and with lacquer coating experienced the most considerable color changes in these staining solutions, with statistically significant differences observed.

The results from Table 2, Table 3 and Table 4 show that staining solutions like red wine and coffee have a greater impact on the color stability of tested resins (Crowntec A2, Crowntec A3, and Denture 3D+) than distilled water. This finding is important for evaluating the color stability of dental prosthetic materials.

**Figure 2:**
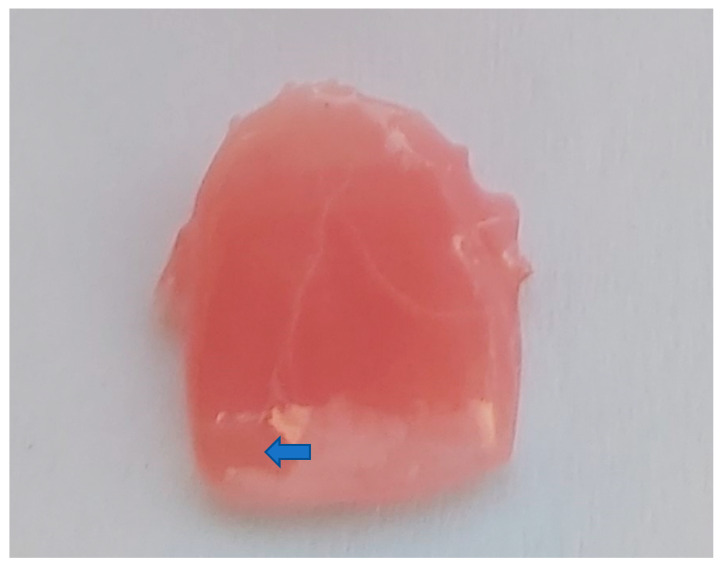
Deture base material Denture 3D +. The surface of the sample is covered with varnish and stored in distilled water. Under the influence of storage, the varnish separates from the resin. Separation is marked with arrows.

The varnish on the surface of the sample peeled off during storage (Figure 2).

**Figure 3:**
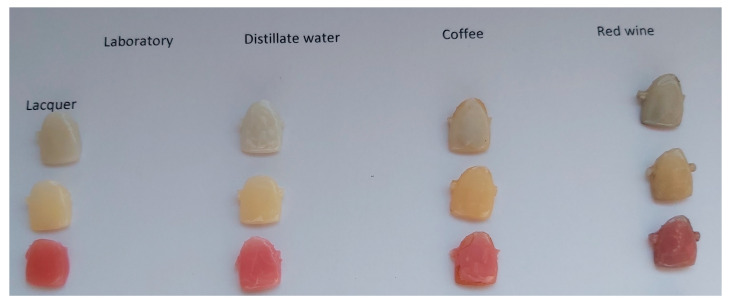
Color changes after storage in different media. The greatest color changes were observed in a test tube stored in red wine.

Tooth color changes in all media; distilled water, coffee, and red wine are shown in Figure 3 for the varnished samples.

**Figure 4:**
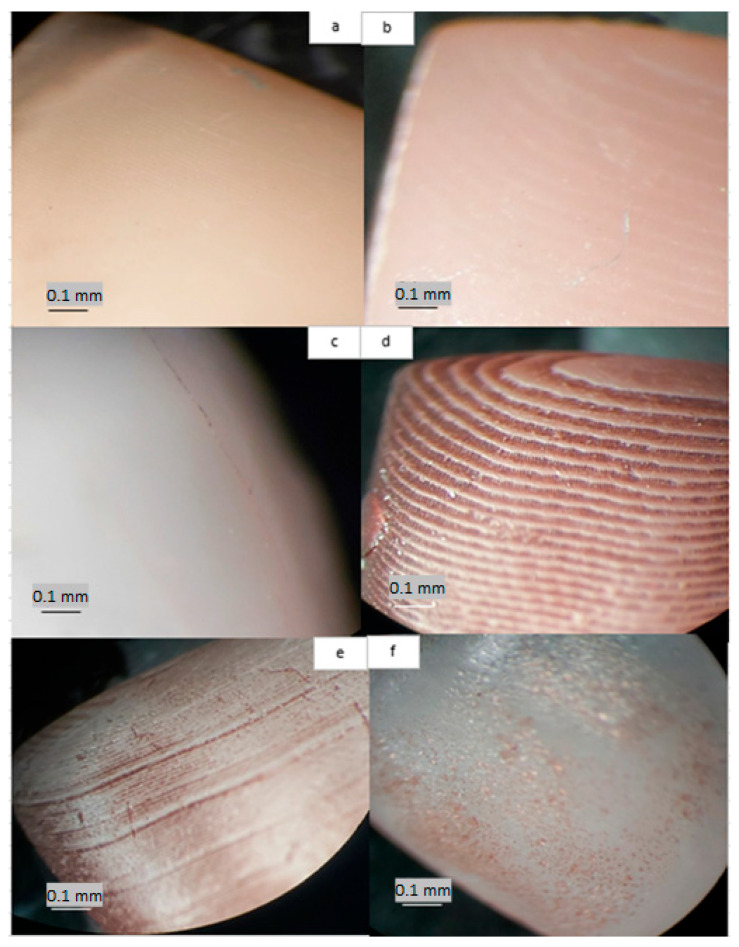
(**a**) Photograph of the tooth surface under a microscope. It is visible that polishing the tooth surface provides less retention for dyes (Crowntech A3); (**b**). Teeth covered with varnish are protected against discoloration, but after a long time, the protecting layer can crack (Crowntech A2); (**c**) Teeth covered with varnish, after storage in distilled water. Lacquer cracks (arrows), and the remnants of dyes begin to accumulate in the gaps (Crowntech A2). (**d**) Teeth’s unpolished surface after storage in red wine, and (**e**) after contact with coffee. Between the individual layers of the material as it was printed are visible dyes (Crowntech A2). (**f**) Sample of A2 color storage in red wine for 60 days, with a polished surface but not enough; a clearly visible region with greater roughness, where the absorption of dyes took place (Magnification 25×).

Samples polished by the traditional method with a pumice stone show the least change in color during storage in staining solutions. (Figure 4a).

If the varnish did not separate from the surface of the materials, then the surface of the artificial teeth is smooth and did not change color (Figure 4b). However, if the surface of the material became contaminated, the varnish began to crack and scratches formed on the surface, which absorb the dyes (Figure 4c).

When comparing the three surface treatments, samples without polishing displayed the most significant color change in red wine and coffee solutions (Figure 4d,e). This indicates that not polishing the surface of prosthetic restorations can lead to higher susceptibility to discoloration when exposed to staining agents. On the other hand, lacquer coating provided better color stability than unpolished samples, but in some cases, it showed more color change than mechanically polished samples. This suggests that whereas lacquer coating offers some protection against staining, mechanical polishing might be more effective in maintaining the color stability of dental prosthetic materials.

Mechanical polishing demonstrated better color stability compared to the other two surface treatments in most cases, making it an effective method for preserving color stability when exposed to common staining agents like red wine and coffee. The polishing procedure must be carried out very carefully, because even the smallest surface irregularities absorb dyes (Figure 4f—red wine).

During the experiments, cracks and peeling of varnish pieces were observed on the test surfaces, as shown in Figure 2 and Figure 4c. Tooth color changes were documented and are displayed in Figure 3, with the most significant color changes observed in the samples stored in red wine.

Microscopic observations of polished tooth surfaces indicated that they were less susceptible to staining than non-polished samples, as illustrated in Figure 4. Very significant changes were observed in Figure 4d,e when the presence of red wine or coffee dye between the individual layers of the unpolished material could be seen.

The results of the tests show that unpolished materials undergo the most significant color change in red wine after 60 days. The second-highest color change occurred in rough samples exposed to coffee. Samples with varnish-protected surfaces exhibited the smallest color changes. However, over time, some surfaces began to experience varnish peeling. The least color change was observed in samples stored in distilled water.

The surface treatment of dental prosthetic materials plays a crucial role in their color stability. Unpolished surfaces are more susceptible to staining, whereas varnish protection can help maintain color but may experience peeling over time. Polished surfaces demonstrate better color stability, but it is essential to ensure adequate polishing to avoid areas with greater roughness that may absorb dyes.

## 4. Discussion

The thesis put forward at the beginning of this study has not been confirmed. The method of preparation of the material affects color stability. Many factors can affect color changes in temporary dental materials, such as polymerization, diet, dyes, oral hygiene, and surface smoothness. The selection of the staining solution and its concentration, as well as the time during which the materials are exposed to the dyeing solution, may also influence the degree of color change and surface topography [23,24,25,26].

Coffee and red wine solutions have long been used as unofficial standards for testing and changing the color of materials in prosthetics [27]. Discoloration of materials can occur externally (deposits on the surface) or internally (when the dye penetrates the inside of the material, monomer composition, and degree of polymerization) [21,24,25].

The NextDent 3D system was tested by Dimirtova et al. in a solution of wine, coffee, Coca Cola and artificial saliva. The authors, as in these studies, observed the greatest color change for the samples stored in red wine for a period of 21 days. The Delta E value was 2.05, which was lower than in these studies, but the storage period of the sample was also much shorter [23].

Materials intended for 3D printing also change their color as a result of sorption and solubility in water, which reduces their translucency is [24,25].

Contact time plays a very important role in the color stability of dental materials [26]. Korean authors have demonstrated in their research that 3D materials exhibit lower color stability compared to materials milled using CAD/CAM technology, in which PMMA discs are polymerized for an extended period under high pressure and at a temperature of approximately 130 °C [27,28,29].

Color stability is also affected by the orientation of the sample when printing. The most accurate restorations are obtained when the sample orientation is 45 degrees (Hada et al.) [28]. In this case, however, the printed crown had rows of layers that came together on the surface, and thus it was easily discolored (Figure 4).

Another potential explanation for the low color stability of 3D printing resins is the degree of double bond conversion of acrylic resins in these materials, which is at a level of 50–80% [18,19,30]. Post-curing processes after printing contribute to higher polymerization rates [31]. The use of red wine to test the color change of dental materials is a common practice in dentistry. Red wine, with its strong pigmentation, can be an effective indicator of how well a dental material is able to resist staining over time. It is often used as a second solution for testing the color stability of dental restorative materials, after coffee. Red wine is also a convenient choice for testing dental materials because it is readily available and easy to use [32,33,34]. However, it is important to note that the staining potential of red wine can vary depending on the type and quality of the wine, as well as other factors such as pH and tannin content [35].

The immersion period and the frequency of exposure to staining solutions are other factors that can influence color stability [36]. Studies have shown that longer exposure to staining solutions, such as red wine, coffee, or tea, can lead to a more pronounced color change in dental materials [11].

The role of the staining solution’s pH on the color stability of dental materials should also be considered. The pH of the staining solution can affect the color stability of dental materials, as it may cause erosion, surface roughness changes, or even dissolution of the materials [37]. It is crucial to understand the interaction between the acidity of common beverages and dental materials, as this knowledge can lead to the development of more resistant and durable dental restorations [38].

In our study, we observed that the surface treatment of dental prosthetic materials plays a crucial role in their color stability. Unpolished surfaces are more susceptible to staining, whereas varnish protection can help maintain color but may experience peeling over time. Polished surfaces demonstrate better color stability, but it is essential to ensure adequate polishing to avoid areas with greater roughness that may absorb dyes.

The development of dental materials with enhanced color stability that can mimic the natural appearance of teeth is of utmost importance in modern dentistry. With the esthetic expectations of patients continuously increasing, there is a growing demand for dental restorations with better color stability, biocompatibility, and mechanical properties [39].

Color stability is an important factor when it comes to the durability and longevity of dental restorations. Dental materials with poor color stability can become discolored over time, resulting in unsightly restorations that do not match the natural appearance of the patient’s teeth. Dental materials with enhanced color stability can help ensure that restorations maintain their original color and appearance, leading to greater patient satisfaction [39]. Biocompatibility is another critical consideration when developing dental materials. Biocompatible materials are essential in ensuring that dental restorations do not cause adverse reactions in patients. Dental professionals must consider the biocompatibility of dental materials to reduce the risk of allergic reactions or infections, leading to better patient outcomes. Mechanical properties such as strength, durability, and resistance to wear are also important when it comes to dental restorations. Dental materials with superior mechanical properties can help ensure the longevity and effectiveness of restorations. Such materials can withstand the forces of biting and chewing, reducing the risk of breakage or cracking, and provide patients with a functional and long-lasting restoration [39]. Researchers are continuously working on improving the properties of dental materials, including the development of new resin-based composites, ceramics, and CAD/CAM materials with better color stability [40,41,42].

Patient education and preventive measures should be underlined in order to minimize the risk of staining in dental restorations. Dental professionals play a crucial role in educating their patients on the proper care and maintenance of their dental restorations. Patients should be informed about the potential effects of staining beverages, such as red wine, coffee, and tea, on the color stability of their restorations, and advised on proper oral hygiene practices and maintenance of their dental work.

Staining beverages can have a significant impact on the color stability of dental restorations, particularly those made from materials such as composite resin or ceramic. These materials can become discolored over time when exposed to pigmented substances, leading to unsightly restorations that do not match the natural appearance of the patient’s teeth. Dental professionals should inform patients about the potential risks of consuming staining beverages and provide recommendations for reducing their consumption or minimizing their impact on dental work [40,43,44,45,46].

Performing research has its limitations. Three-dimensional printing materials from one manufacturer, NextDent, were tested using one printer and one type of light oven. As is well known, the degree of curing of light-curing materials may vary depending on the devices used, both the 3D printer and the light oven, which may affect the results obtained [28,29]. Therefore, further research into other materials and curing equipment is needed.

The effect of the type of resin used in 3D printing on the color stability of dental restorations is an area that requires further research. Different resins have different characteristics, including composition, degree of polymerization, and physical properties, which can affect their susceptibility to discoloration and staining [47]. A better understanding of the relationship between resin type and color stability can help dentists choose the most appropriate materials for their patients, leading to more esthetically pleasing and longer-lasting restorations.

In the context of color stability, it is also important to consider the role of saliva and its interaction with dental materials. Saliva is essential in the oral environment, providing natural protection against discoloration and maintaining the integrity of dental restorations. The complex composition of saliva, including its buffering capacity, enzymes, and proteins, can affect the surface properties and color stability of dental materials over time [48,49]. Further research into the interaction between saliva and dental materials may provide valuable information to develop more effective strategies to improve the color stability of dental restorations in clinical settings.

The development of advanced dental materials with improved color stability and stain resistance is an ongoing area of research. Innovations in materials science, such as the use of nanoparticles, antimicrobial agents, or advanced surface treatments, may offer potential solutions for improving the color stability of dental restorations [50,51]. In the future, research into new methods to improve the esthetic properties and durability of dental materials should be continued, providing patients with the highest quality of care and satisfaction with dental treatment [52,53].

In this work, a more comprehensive study of factors affecting the color stability of restorations was performed, building on previous research in this area. For example, Dimitrov et al. [24] focused on the color stability of restorations fabricated using the NextDent 3D system, but our study goes beyond the scope of their work by examining a wider range of restorations and considering different surface preparation methods.

While Dimitrov et al. [24] primarily investigated the susceptibility of the NextDent 3D system to discoloration, our work delves into the effect of resin type on color stability. By evaluating different resins and their susceptibility to staining and discoloration, we aim to offer dentists a better understanding of the most appropriate materials to use, which will ultimately contribute to the development of more esthetically pleasing and durable restorations.

Our study also investigates the role of saliva and its interaction with dental materials, an aspect that has been largely overlooked in previous studies, including Dimitrov et al. [24]. By analyzing the complex relationship between saliva and dental materials, we hope to provide valuable information for the development of more effective strategies to improve the color stability of dental restorations in clinical settings.

In the study conducted by Almejrad et al., the color stability of 3D-printed interim restorations with different surface treatments was evaluated after being immersed in various staining solutions, such as artificial saliva, tea, coffee, and wine for six months. Using a laboratory scanner, CAD/CAM software, and 3D-printing technology, 80 abutment teeth and interim restorations were produced from tooth-colored photopolymerizing resin and randomly assigned to either a Polish or Optiglaze treatment group. The samples were then divided into four subgroups based on their immersion liquid. Color measurements were taken pre- and post-immersion, and two-way ANOVA was conducted to assess the impact of surface treatment, immersion liquid, and their interaction on color change (ΔE) after six months. Results showed significant effects of surface treatment, immersion liquid, and their interaction on ΔE, with red wine causing the most discoloration. The application of a nanofilled, light-polymerizing protective coating was found to significantly reduce discoloration from chromogenic beverages, particularly coffee, suggesting its usefulness for extended intraoral service of 3D-printed interim restorations [54].

Other studies have investigated the color stability of dental restorations made from 3D-printing resins and conventional CAD/CAM blocks, focusing on the impact of various colorants and storage durations. It has been found that 3D-printing resins exhibited significantly higher discoloration than CAD/CAM blocks when exposed to grape juice, coffee, curry, and distilled water over 2, 7, and 30 days. The findings highlight the importance of considering discoloration when using 3D-printing resins for dental restorations [32].

In research performed by Scotti et al., the physical and surface properties of a 3D-printed resin were compared with those of materials traditionally used for interim dental restorations. Three different materials were tested for color change, flexural strength, Knoop hardness, and surface roughness: a 3D-printed resin, an autopolymerizing interim material, and a composite resin. The results revealed that the composite resin exhibited the highest values for flexural strength and hardness, followed by the 3D-printed resin, whereas the autopolymerizing interim material demonstrated the lowest values for both tests. In terms of surface roughness, the 3D-printed resin showed similar values to the autopolymerizing material, but higher values than the composite resin. However, the 3D-printed resin displayed the most significant color variation over time, raising concerns about its color stability for long-term use. Despite this, the 3D-printed composite resin showcased adequate mechanical and surface properties, suggesting its potential as a cost-effective alternative for interim restorative materials in dentistry [55].

## 5. Conclusions

1. This study highlights the importance of proper curing, surface polishing, and the potential limitations of using varnish for 3D-printed dental restorations.
2. The results indicate that materials intended for 3D printing must undergo complete curing to minimize color change and ensure long-lasting esthetic results. Moreover, the surface of the material after curing should be meticulously polished using traditional techniques to eliminate any surface imperfections that may lead to localized discoloration over time.
3. Whereas varnish application might be useful for temporary restorations, its long-term use may not be ideal for 3D-printed restorations. This is due to the potential for the varnish to crack and create fissures on the surface, allowing dyes to penetrate and negatively impact the esthetic appearance of the restoration. The disparity in flexibility between the varnish resin and the 3D printing material may contribute to this issue.

## Figures and Tables

**Table 1:** Composition of testing resins.

Material	Composition
Denture 3D+	Ethoxylated bisphenol A dimethacrylate => 75%,
Crowntec A3 and A2	7,7,9(or 7,9,9)-trimethyl-4,13-dioxo-3,14-dioxa-5,12-
	diazahexadecane-1,16-diyl bismethacrylate 10–20%
	2-hydroxyethyl methacrylate 5–10%
	Silicon dioxide 5–10%
	diphenyl(2,4,6- trimethylbenzoyl)phosphine oxide 1–5%
	Titanium dioxide < 0.1%

**Table 2:** Color change of Crowntec A2 color over time in different media.

	ΔE 14 Days	ΔE 30 Days	ΔE 60 Days	*p* Value
LW	0.42 ± 0.03	0.54 ± 0.10	0.93 ± 0.22	*p* < 0.5 *
LC	0.50 ± 0.14	1.10 ± 0.23	1.37 ± 0.41	
LR	0.70 ± 0.02	1.05 ± 0.08	1.59 ± 0.11	*p* < 0.01 *
NpW	2.94 ± 0.51	3.36 ± 0.49	3.65 ± 0.30	
NpC	3.25 ± 0.23	9.70 ± 0.34	11.54 ± 0.62	*p* < 0.01 *
NpR	3.34 ± 0.26	16.27 ± 0.21	18.19 ± 0.16	*p* < 0.01 *
PW	0.36 ± 0.02	1.44 ± 0.06	3.59 ± 0.42	*p* < 0.01 *
PC	2.02 ± 0.28	3.32 ± 0.30	5.00 ± 0.49	*p* < 0.01 *
PR	2.18 ± 0.21	12.59 ± 0.82	13.61 ± 0.42	*p* < 0.01 *

* Statistically significant *p* values are given. LW—sample covered with lacquer and stored in distilled water, LC—sample covered with lacquer and stored in coffee solution, LR—sample covered with lacquer and stored in red wine, NpW—sample non-polished after polymerization and stored in distilled water, NpC—sample non-polished and stored in coffee solution, NpR—sample non-polished and stored in red wine. PW—sample polished and stored in distilled water, PC—sample polished and stored in coffee solution, PR—sample polished and stored in red wine.

**Table 3:** Color change of Crowntec A3 color over time in different media.

	ΔE 14 Days	ΔE 30 Days	ΔE 60 Days	*p* Value
LW	0.65 ± 0.06	0.69 ± 0.25	0.75 ± 0.16	
LC	0.54 ± 0.08	1.67 ± 0.36	1.97 ± 0.23	
LR	1.33 ± 0.26	2.55 ± 0.53	3.45 ± 0.30	*p* < 0.01 *
NpW	0.45 ± 0.06	0.73 ± 0.09	0.94 ± 0.51	
NpC	2.72 ± 0.21	5.45 ± 0.43	6.50 ± 0.28	*p* < 0.01 *
NpR	4.77 ± 0.29	5.85 ± 0.20	6.64 ± 0.33	
PW	0.50 ± 0.10	1.01 ± 0.10	1.11 ± 0.36	
PC	2.64 ± 0.31	3.34 ± 0.36	3.61 ± 0.42	
PR	3.33 ± 0.33	8.02 ± 0.44	10.94 ± 0.23	*p* < 0.01 *

* Statistically significant *p* values are given. LW—sample covered with lacquer and stored in distilled water, LC—sample covered with lacquer and stored in coffee solution, LR—sample covered with lacquer and stored in red wine, NpW—sample non-polished after polymerization and stored in distilled water, NpC—sample non-polished and stored in coffee solution, NpR—sample non-polished and stored in red wine. PW—sample polished and stored in distilled water, PC—sample polished and stored in coffee solution, PR—sample polished and stored in red wine.

**Table 4:** Color change of Denture 3D+ color over time in different media.

	ΔE 14 Days	ΔE 30 Days	ΔE 60 Days	*p* Value
LW	0.63 ± 0.19	1.33 ± 0.42	1.36 ± 0.27	
LC	0.76 ± 0.13	2.30 ± 0.26	2.51 ± 0.40	
LR	0.83 ± 0.18	2.50 ± 0.32	3.48 ± 0.13	*p* < 0.01 *
NpW	0.35 ± 0.18	1.51 ± 0.68	1.80 ± 0.14	
NpC	2.10 ± 0.27	4.76 ± 0.26	5.59 ± 0.49	*p* < 0.01 *
NpR	4.88 ± 0.33	9.04 ± 0.56	11.00 ± 0.28	*p* < 0.01 *
PW	0.73 ± 0.11	1.36 ± 0.39	1.53 ± 0.27	
PC	1.05 ± 0.12	2.46 ± 0.24	2.78 ± 0.25	
PR	2.45 ± 0.34	2.45 ± 0.34	4.21 ± 0.31	

* Statistically significant *p* values are given. LW—sample covered with lacquer and stored in distilled water, LC—sample covered with lacquer and stored in coffee solution, LR—sample covered with lacquer and stored in red wine, NpW—sample non-polished after polymerization and stored in distilled water, NpC—sample non-polished and stored in coffee solution, NpR—sample non-polished and stored in red wine. PW—sample polished and stored in distilled water, PC—sample polished and stored in coffee solution, PR—sample polished and stored in red wine.

## Data Availability

Data sharing is not applicable to this article.
